# Loners Have It: Insights on Breeding and Non‐Breeding Individuals of Sloth Bears in Central India

**DOI:** 10.1002/ece3.74231

**Published:** 2026-09-17

**Authors:** Sankarshan Chaudhuri, Supratim Dutta, Ramesh Krishnamurthy

**Affiliations:** ^1^ Department of Landscape Level Planning and Management Wildlife Institute of India Dehradun Uttarakhand India; ^2^ Faculty of Forestry University of British Columbia Vancouver British Columbia Canada

**Keywords:** behavioural ecology, infanticide, *Melursus ursinus*, multistate occupancy, Panna Tiger Reserve, sexual segregation

## Abstract

Population persistence is critically dependent on the reproductive biology of organisms, which often remains poorly understood, especially for solitary and elusive carnivores. Exhibiting intraspecific reproductive state segregation, as a part of breeding biology, indicates avoiding infanticides and/or reducing resource competition. Sloth bear ( *Melursus ursinus* ) is the most common ursid in the Indian Subcontinent, yet knowledge of their breeding biology concerning intraspecific interaction is scanty. We assessed the occupancy and temporal activity of sloth bears for two states related to their breeding, that is, lone adults and females with offspring, aiming to understand the spatio‐temporal segregation by using data obtained from camera trap surveys (2018–2021) in Panna Tiger Reserve, central India. Dynamic and single‐season multistate occupancy models indicated spatial segregation between females with offspring and lone adults, as the former occupied hotter, rugged terrains, while lone adults preferred mixed forests and cooler habitats. However, during summer, seasonal forage availability perhaps contributed to less (but existing) spatial avoidance shown by females with offspring. We found fine‐scale temporal segregation between the two states, regardless of the seasons. Such spatio‐temporal interactions may be driven by the protection of cubs from infanticide by conspecifics or additively with the reduction in foraging competition, which probably came with a physiological cost to the females with offspring. Our study is among the first to quantitatively compare indicators of occupancy between lone adults and females with offspring, as a significant part of sloth bears' breeding biology. Demonstrating such spatio‐temporal segregation patterns in reproductive states represents a promising approach to advancing our ecological understanding of population structure and fitness outcomes in sloth bears.

## Graphical abstract

Dynamics of breeding biology, such as intraspecific sexual segregation, indicate avoidance of infanticide and/or reduced resource competition, which are reported for some bear species but are lacking for Sloth bears ( *Melursus ursinus* ). We found that mother bears with cubs segregate themselves from lone adults spatio‐temporally by avoiding resource‐rich areas, in addition to moderate temporal partitioning. This perhaps incurs a fitness trade‐off for breeding females, which may contribute to shaping the population structure of sloth bears, and is a matter of further research.

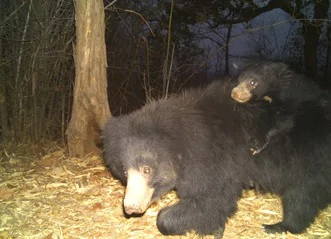

## 1 Introduction

A thriving population is critically dependent on one of the vital life history parameters, that is, reproduction. While reproduction output or success indicates the population's viability, breeding success or failure could be affected by a plethora of biotic and abiotic factors; however, the complex nature of such mechanisms remains poorly understood. Predation is one of the major evolutionary forces that can negatively impact the fitness of an individual, often through the death of its offspring (Ferrari et al. 2009; Lima and Bednekoff 1999), and the alteration of parental care strategies can minimise the mortality risk from adult unrelated males to ensure the survival of the offspring (de Moraes et al. 2020). Reproduction is closely interlinked with resource availability (Nichols et al. 2012), and differential patterns of resource utilisation in the presence of con‐ and heterospecifics indicate complex species interactions at play in determining offspring survival (Brown et al. 2001; Lima and Bednekoff 1999; Main et al. 1996). Hence, understanding reproductive success requires knowledge of the complex behavioural patterns adopted by females to avert the risk of predation of their offspring.

In most cases, space use patterns are not explicitly addressed with respect to the demography (such as females with offspring and lone adults in the present study) but are presented at a species level (Fernández et al. 2003; Ramesh et al. 2012; Rather et al. 2021). With this scarce knowledge, it often remains a matter of concern how breeding success could be linked to the spatial patterns of landscape features and, additionally, temporal behaviour, especially when intraspecific competition for resources and mate choice is persistent (Forsgren et al. 1996; Orgeret et al. 2021). However, acquiring the data from the field to address this critical yet universal ecological concern could be notoriously difficult, especially when the target species is solitary and elusive (Steyaert et al. 2013). In mammals, reproductive females often segregate themselves (with their offspring) from the conspecifics, including adult males and females (Ruckstuhl 2007; Ruckstuhl and Neuhaus 2000; Steyaert et al. 2013; Wielgus and Bunnell 2000). Such behaviour is even more prominent among large solitary carnivores to avoid infanticides and maximise the fitness of the offspring (Keehner et al. 2015; Wielgus et al. 2001; Wielgus and Bunnell 2000). The non‐parental infanticide (NPI) is generally linked to the reduction in foraging competition or the induction of adaptive male reproduction by killing unrelated offspring (sexually selected infanticide, SSI) (Hrdy 1979; Hrdy and Hausfater 1984). The latter is more common in sexually dimorphic polygynous carnivores (Packer and Pusey 1984) with prolonged parental care (Pusey and Packer 1994), but testing these hypotheses comprehensively or assessing the cause of such behaviours poses a challenge in the wild.

**FIGURE 1:**
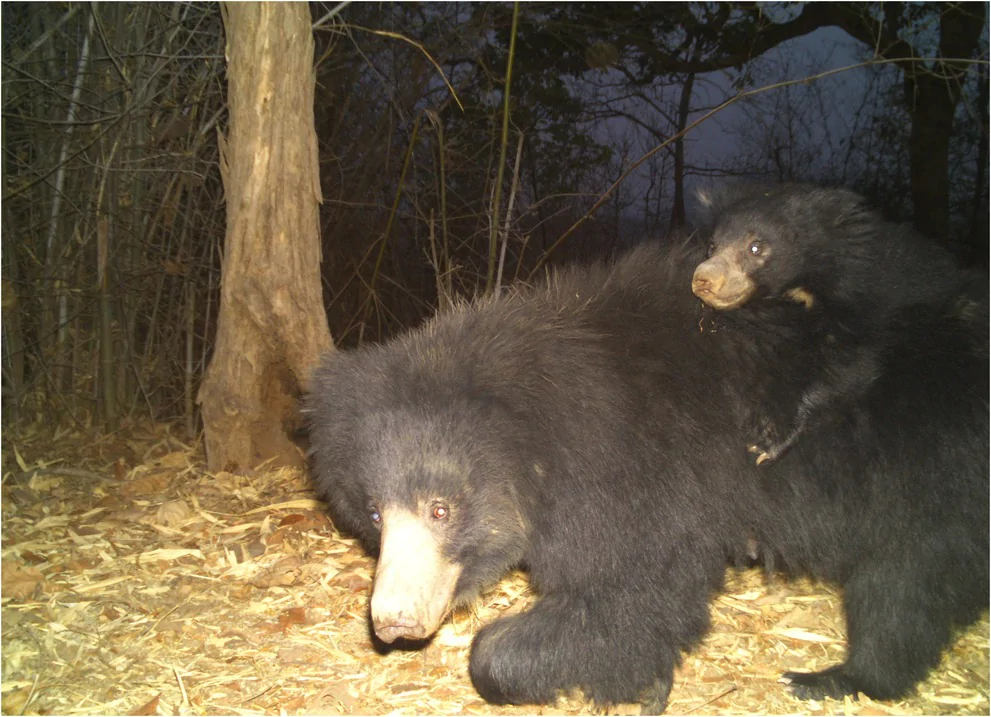
An adult female sloth bear, carrying its cub (< 1‐year‐old) on its back, in Panna Tiger Reserve, Madhya Pradesh, central India.

In the case of SSI, eliminating the unrelated offspring facilitates the perpetrator in increasing their reproductive success (Hrdy 1979). However, mammals exhibiting such phenomena often experience severe male–male competition with short reproductive tenure (Hiraiwa‐Hasegawa 1988). Also, in a polygynous mating system, SSI can be either prominent among species where females show a flexible reproductive system and become receptive shortly after the loss of offspring or among strictly seasonal breeders when the infants will mostly be the victims, especially during the mating season (Ebensperger 1998; Hrdy 1979). On the other hand, if infanticide is a result of curbing the competition for resources, the vulnerability and size of the offspring will be the determining factors, and the perpetrators could be both adult males and females (Hrdy and Hausfater 1984). Although rare, incidences of infanticides by females have also been recorded in carnivores, which were thought to be primarily a result of decreasing the forage competition by the perpetrator (Lukas and Huchard 2019; Roex et al. 2023). Nonetheless, being one of the intense selective pressures in the mammalian reproductive system, the counterstrategies to prevent infanticide have also evolved among females as a form of adaptive change (Agrell et al. 1998; Ebensperger 1998). Such strategies include physiological changes, behavioural changes, and, lastly, changes in ecological requirements, such as spatial and/or temporal segregation in the presence of unrelated and potential infanticidal individuals (Agrell et al. 1998; Ebensperger 1998; Wielgus and Bunnell 1994, 2000). A few pieces of evidence of such spatio‐temporal segregation can be found in past studies on social carnivores such as lions (Packer and Pusey 1983, 1984) and solitary carnivores such as mountain lions (Keehner et al. 2015). Long‐term studies showed that ursids, especially brown bears, exhibit intraspecific competition in the form of NPI, committed by both sexes but mainly as a male reproductive strategy, and females developed counterstrategies to avert the risk of offspring mortality (Ben‐David et al. 2004; Steyaert et al. 2012, 2013; Swenson et al. 1997, 2001; Wielgus and Bunnell 1994, 2000). Brown bears were also reported to exhibit SSI, but it varied with their geographical distributions (Ben‐David et al. 2004; Craighead et al. 1995; Miller et al. 2003). While brown bears were one of the most studied ursids, especially in the context of their breeding and cub survival, such knowledge is minimal for other ursids, such as sloth bears ( *Melursus ursinus* ). Sloth bears are medium‐sized ursids with a body weight that ranges from 80 to 150 kg for males and 60–100 kg for females (Prater 1965). They inhabit a wide range of habitats in lower‐elevation areas (< 1000 m) across the Indian Subcontinent, with a strong association with deciduous forests (moist and dry) (Dharaiya et al. 2016; Garshelis, Joshi, Smith, and Rice 1999; Yoganand 2005; Yoganand et al. 2006). Sloth bears are generally considered habitat generalists but show some affinity to certain forest or habitat types such as miscellaneous forests in central India, alluvial grasslands in the Terai Arc region, and deciduous patches in southern India, possibly due to seasonal forage availability (Garshelis, Joshi, and Smith 1999; Chaudhuri, Rajaraman, et al. 2022; Ramesh et al. 2012). This species is primarily nocturnal and crepuscular, with a much smaller yet variable (ranging from ~2 to ~84 km^2^) home range compared to other ursids (Joshi et al. 1995; Yoganand 2005; Ratnayeke et al. 2007; Ramesh et al. 2013; Chaudhuri, Bandyopadhyay, et al. 2022). Sloth bears exhibit an omnivorous diet, mainly comprised of insects and seasonal fruits (Rather et al. 2020). In many parts of their distribution (including the present study area), they are notably involved in human‐wildlife conflict, which poses a conservation challenge (Rajpurohit and Krausman 2000; Dhamorikar et al. 2017; Singh et al. 2018). However, like other bears, sloth bears are known to be solitary and polygynous but do not exhibit territoriality (Joshi et al. 1999; Prater 1965). So far, only one study (by Joshi et al. 1999) aimed to understand the breeding biology of sloth bears intensively, and the authors indicated some degree of avoidance towards conspecifics shown by females with offspring (Figure 1), perhaps due to the safety of their cubs, but couldn't tease apart their fine‐scale segregation strategies. Also, this single study could not produce strong evidence of NPI in sloth bears but acknowledged undetected cubs' mortality in the population. With such a dearth of knowledge and without further rigorous study, it remains obscure for us to understand how intraspecific competition in the form of NPI (SSI and competition for resources) plays a role in evolving counterstrategies by female sloth bears to prevent offspring mortality. However, it is logical that minimising the risk of infanticide or intraspecific competition could be a function of the habitat where those competing or infanticidal conspecifics occur rather than their density or presence (Boinski et al. 2003), and understanding such dynamics of habitat segregation would be a noteworthy contribution to the behavioural ecology of the study species.

In this paper, we attempted to elucidate whether any sexual spatio‐temporal segregation between female sloth bears with offspring and lone adults exists in a dry deciduous forest of central India, using hierarchical occupancy models under a multi‐season camera‐trapping framework. We identified the potential environmental factors (i.e., habitat, season, temperature) that govern the occupancy dynamics of female sloth bears with their offspring across seasons over multiple years, and examined the differences in occupancy and diel activity patterns between lone adults and females accompanied by offspring. While such behavioural patterns are poorly documented in sloth bears, insights from other carnivores, including ursids, revealed that such demographic classes may indicate differential use of space and time, either for the safety of offspring or to reduce resource competition. The present study, therefore, aimed to assess if these similar patterns are also evident in sloth bears, and accordingly, we expected to detect any spatio‐temporal differences between lone adults and females with offspring in this central Indian landscape.

## 2 Materials and Methods

### 2.1 Study Area

**FIGURE 2:**
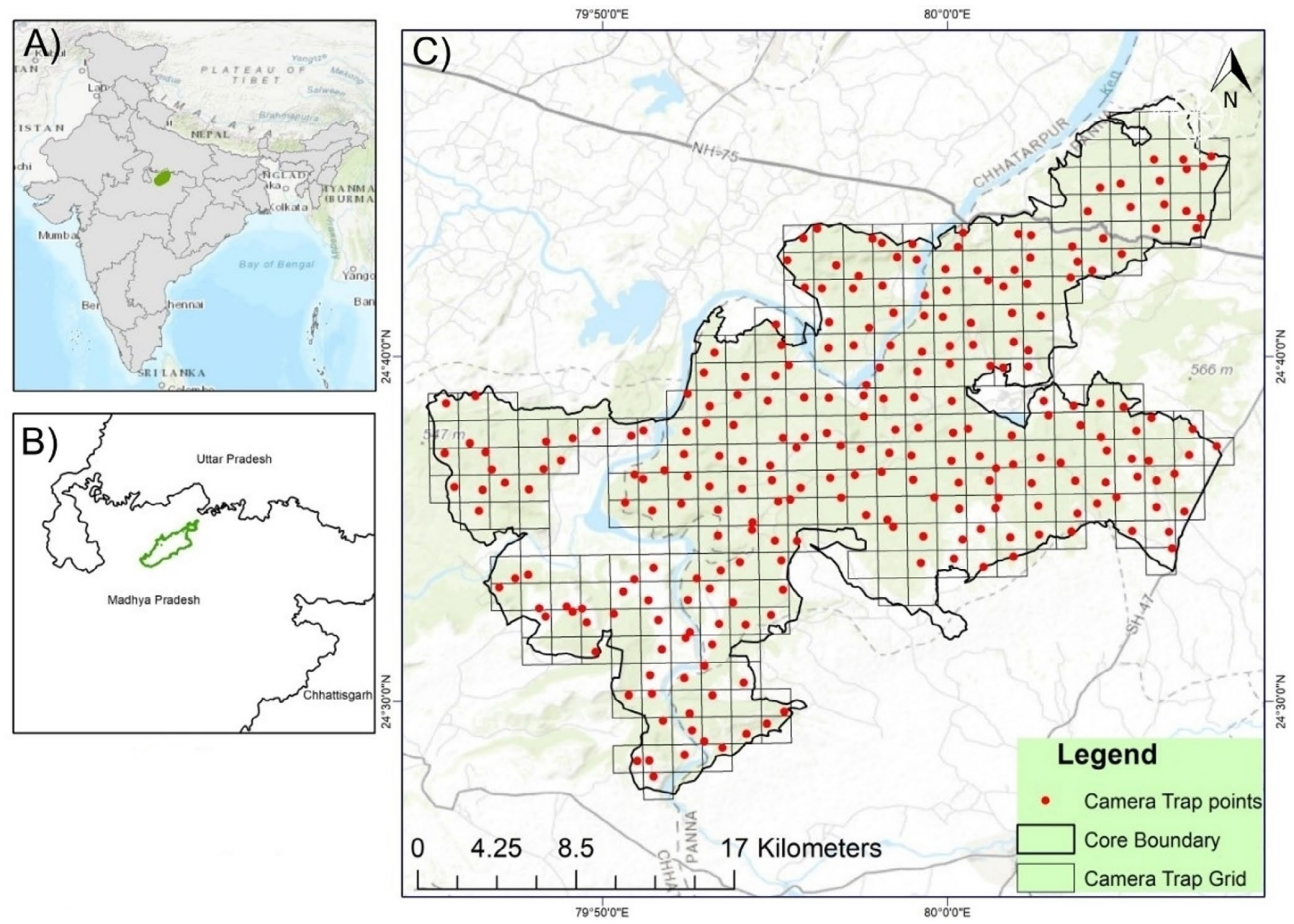
Location of the core zone of Panna Tiger Reserve, situated in Madhya Pradesh, central India, and the locations of camera traps deployed in 2 km^2^ grids during 2018–2021.

Panna Tiger Reserve (PTR) is situated in the Vindhya Hill range of central India, between 24° 27′ and 24° 46′ N latitudes, and 79° 45′ and 80° 09′ E longitudes (Figure 2). PTR comprises an inviolate core zone of 542 km^2^ (the intensive study area) and a surrounding multi‐use buffer zone of 1032 km^2^, thus encompassing an area of 1574 km^2^. The terrain varies from flat areas to steep escarpments, including three major plateaus with an elevation range from 164 to 555 m. Ken, the only perennial river, flows through PTR and divides it into the eastern and western parts. The primary forest type is tropical dry‐deciduous (Champion and Seth 1968). The subtropical dry climate of PTR has three different seasons, i.e., summer (Mean maximum temperature is ~45°C between March and June, relative humidity 15%–20%), monsoon (Average monthly rainfall between July and October is 1100 mm, relative humidity > 70%–90%) and winter (Mean minimum temperature is ~5°C between November and February, relative humidity 15%–34%) (Jayapal et al. 2007). Some of the major tree species are Teak ( *Tectona grandis* ), Tendu (*Diospyros melanoxylon*), and Kardhai (*Anogeissus pendula*). Under the tropical dry‐deciduous forest formation, major vegetation/habitat types of PTR are mixed, teak mixed, bamboo mixed, teak forest, riverine, *A. pendula* forest, scrubland and grassland. In addition to the focal species, PTR supports several large and mesocarnivores, including Tiger ( *Panthera tigris* ), Leopard ( *Panthera pardus* ), Striped Hyena ( *Hyaena hyaena* ), Asiatic Wild Dog ( *Cuon alpinus* ) and Grey Wolf ( *Canis lupus* ). Chital ( *Axis axis* ), Sambar ( *Rusa unicolor* ), Wild Pig ( *Sus scrofa* ), Nilgai ( *Boselaphus tragocamelus* ), Chinkara ( *Gazella bennettii* ) and Chousingha ( *Tetracerus quadricornis* ) are the most important herbivores. The core zone is highly protected but includes three villages, while the buffer zone holds 63 villages, which makes it a human‐dominated multi‐use landscape. While anthropogenic exposure is limited in the core zone, the buffer zone including the surrounding landscape, experiences a moderately high level of conflict with sloth bears (~6 attacks on humans/year) in addition to other large carnivores and herbivores (WII 2022).

### 2.2 Field Methods

We conducted systematic camera trap surveys from 2018 to 2021 in the core zone of PTR, following the All India Tiger Estimation (AITE) (Jhala et al. 2020). During the period mentioned above, a total of six seasons were covered, that is, winter 2018, winter 2019, summer 2019, winter 2020, summer 2020 and winter 2021 (hereafter, ‘seasons’ for winters and summers between 2018 and 2021); however, camera trapping was restricted during the monsoon due to logistical constraints (less accessibility) associated with heavy rainfall. We deployed two motion‐censored camera traps (Cuddeback C1 and Cuddeback Attack; www.cuddeback.com) opposite each other in each 2 km^2^ grid, and season‐wise deployed camera traps varied between 243 and 254 during 2018–2021 (Figure 2). During the camera trap survey in winter 2020, camera stations in 69 locations continued to be functional during April, and the data gathered during this period (i.e., from March till April, after the winter) was assigned for summer season. We deployed camera traps on forest roads or trails at a height of 30–40 cm from the ground to increase the photographic detection of sloth bears and other carnivores (Jhala et al. 2020; Pease et al. 2016). The interval between two consecutive photographs was kept at 5 s in order to avoid detection failure of sloth bears, especially cubs or yearlings accompanying their mother. The average distance between the two camera trap stations was 1056.6 m, increasing the detection probability of sloth bears and covering different habitat types. We attempted to keep camera traps functional for 30 to 40 days on a 24‐h basis, and camera traps were periodically (once a week) checked for batteries and memory cards.

### 2.3 Analytical Methods

#### 2.3.1 Dynamic and Single‐Season Multistate Occupancy Framework

Upon retrieval of camera traps, photographic detections of sloth bears were segregated into two categories: lone adults (could be males or females) and females accompanied by offspring, which included cubs (< 1‐year‐old) and dependent yearlings (1.5–2.5 years old) (Joshi et al. 1999; Wakkinen and Kasworm 2004). After emerging from the natal dens, mother bears carry their cubs on their back for at least 6 months (Prater 1965; Yoganand et al. 2013). While the identification of cubs was relatively unambiguous due to their close association with their mothers, there was a risk of misclassifying yearlings as dependents if they were not photo‐captured with their mothers. The short trigger time (5 s) of camera traps enabled us to detect females along with yearlings in most cases. Additionally, we could identify those individuals correctly based on the body size from the consecutive (after 15 s of the first detection) photographs of female bears and yearlings. Initially, we considered a 24‐h period as an occasion to construct the detection history, which is a common practice for camera trap‐based occupancy studies (Goldstein et al. 2024). During this period, the following detections will be recorded: ‘0’ if no bear was detected, ‘1’ for single bear(s) or lone adult(s), and ‘2’ for the female(s) with offspring. Since multiple individuals (lone adults and females with offspring) could be detected during the 24‐h period, we classified the detection as ‘2’ for that particular occasion (Evans et al. 2024). Depending on the number of active camera trap days/season, camera trap records (30 consecutive 24‐h occasions per season) were collapsed into 5–6 sampling occasions, each occasion containing a 5‐day block. Within each block, multiple detections of different states of individuals were pooled together into a single observation. For example, a 5‐day sequence such as 00110 was recorded as 1, and 00002 as 2; where both states were detected within a block, the occasion was classified as state 2 following a hierarchical definition. The choice of the 5‐day detection window to define the sampling occasion for the final analyses was suited for elusive, long‐ranging species (like sloth bears) to reduce the movement‐related bias arising from the ‘temporary emigration’ from a site, and to increase the temporal independence between detections (Sollmann 2018). Such a reduction in temporal replicates/occasions also helps reduce zero inflation and overdispersion (variance > mean) (Penjor et al. 2019), in addition to addressing the effects of autocorrelations for our given study design and study species (Goldstein et al. 2024). Hence, we grouped each season's detection history into six sampling occasions (except for winter 2018, for which the sampling occasion was 5), and we combined them to construct the multi‐season detection history for the subsequent analysis.

Multistate occupancy models assume three states of individual occurrences in a site, ‘1’ being the presence of single individuals or lone adults (occupancy state psi, Ψ), ‘2’ being the state characterised by the presence of offspring with females (a sign of breeding state; occupancy state R) and ‘0’ being the absence of state 1 and 2, that is, unoccupied by the species (Nichols et al. 2007). This model explicitly accounts for the state‐specific detection probabilities, that is, *p*1 for single individuals/lone adults when the occupancy state is 1, and *p*2 for any individual(s) (single or with offspring), given that the occupancy state is 2 for that site (Nichols et al. 2007). Additionally, multistate models could handle the misidentification of state 2 by calculating the observation probability (δ) of detecting offspring if the site is occupied by females with offspring (Nichols et al. 2007). The dynamic multistate occupancy framework additionally computes the change of state (from 0 to 1, 0 to 2, 1 to 2, 2 to 1, 1 to 1, 2 to 2) or transition probabilities (Cpsi or CΨ for state 1 and CR for state 2) if the data are obtained from multiple seasons (Mackenzie et al. 2009). We used the dynamic multistate model to estimate the occupancy probabilities (for state 1: Ψ, CΨ; for state 2: R, CR) of sloth bears as a function of occupancy covariates during 2018–2021, while also modelling the detection and observation probabilities (*p*1, *p*2 and δ) as a function of detection covariates. We prepared raster layers of environmental covariates (forest types, tree cover, slope, topographic position index, land surface temperature, distance to water sources and villages) at two different scales, that is, 30 m and 1 km, to model the detection and occupancy probabilities, respectively (details in Table S1). Additionally, camera trap efforts, as the mean number of days cameras were active during 2018–2021 and seasons (winter and summer, a categorical covariate), were used to model the detection and observation probability of sloth bears. While seasons influence sloth bears' detection in general (Chaudhuri, Bandyopadhyay, et al. 2022), females with cubs are more detected during the summer after emerging from their natal dens, thus increasing their overall detectability (Joshi et al. 1999). However, dependent yearlings could be more frequently observed with females during winter. For strictly territorial organisms, assigning the highest state (State 2) at a site generally excludes the probability of state 1. However, despite being solitary, sloth bears show limited territoriality with extensive range overlap (Joshi et al. 1999; Yoganand 2005); hence, the assumption does not hold true for our study species. Since we expected a range overlap between the two states' individuals, we could not completely rule out the possibility that state 2 individuals would potentially mask the presence of lone adults to some degree. Hence, in addition to the aforementioned detection covariates, we used the detections of females with offspring across the seasons to model the *p*1, in order to assess any influence on the detection of lone adults.

The dynamic multistate occupancy model enables us to tease apart the occupancy for lone adults and females with offspring as a means of transition probabilities over the seasons (Mackenzie et al. 2009). We believe that the derived transition probabilities from multi‐season models will be particularly useful in capturing the fine‐scale dynamics of differential space use shown by sloth bears. We refrained from interpreting transition states, that is, state 2 to 1 and vice versa, due to the probable presence of individuals from both states and to avoid model overparameterization. Instead, we were interested in the dynamic nature of occupancy change from unoccupied to occupied (by lone adults or females with offspring, i.e., state 0–1 or 0–2), and their consistent use of those occupied sites (state 1–1 and state 2–2). We also relaxed the assumption of site independence in occupancy modelling and interpreted the occupancy as the probability of space use (MacKenzie et al. 2017). Prior to model development, we standardised all continuous covariates by calculating *z*‐scores, checked pairwise correlations to curb multicollinearity issues, and refrained from using correlated covariates (threshold was set to |*r*| = ±0.5) together in the modelling.

We first modelled the observation (*δ*) and detection probabilities (*p*1 and *p*2) while keeping the other occupancy parameters constant, following a sequential‐by‐sub‐model framework (Evans et al. 2024; Morin et al. 2020). Based on the information‐theoretic approach, we selected the best models depending on the lower Akaike Information Criterion (AIC) values (ΔAIC < 2) and the ecologically realistic relationships between the parameters of interest and covariates (Burnham and Anderson 2002). We separately modelled *δ*, *p*1 and *p*2 with specific covariates and incorporated a seasonal effect (difference) for each parameter. We finally constructed the best candidate models for the observation and detection probabilities based on the most influential covariates identified for each parameter of interest (Morin et al. 2020). Once the detection sub‐model was finalised, we constructed models for the occupancy and transition probabilities (Ψ, CΨ0, CΨ1, R, CR0 and CR2), retaining the selected detection sub‐models. Similarly, we individually modelled each parameter of interest with occupancy covariates while holding other parameters constant and subsequently developed the final candidate model set for occupancy and transition parameters. We followed the same strategy for developing single‐season multistate models for summer seasons in 2019 and 2020. Due to an insufficient number of seasons (two summers), we adopted a single‐season framework instead of a multi‐season to assess any space use difference between lone adults and females with offspring during summer. Past research suggested that sloth bears' space use may vary across seasons, likely driven by seasonal diet shifts, but is relatively consistent within the same season (Chaudhuri, Rajaraman, et al. 2022; Desai et al. 2026; Palei et al. 2020; Rather et al. 2020; Seidensticker et al. 2011). We used Programme PRESENCE (Hines 2006) for the dynamic and single‐season multistate occupancy analyses.

#### 2.3.2 Temporal Activity Pattern Analysis

Sloth bears are known to be nocturnal and crepuscular (Ramesh et al. 2013), but could also remain active during the daytime, especially subadults and females with offspring, possibly to avoid either large carnivores or adult conspecifics (Joshi et al. 1999). As it is not biologically plausible for sloth bears (especially females with offspring) to be always active throughout the day, we hypothesise that they could still show temporal separation from lone adults, perhaps to reduce the foraging competition and/or SSI. Hence, to assess the activity pattern of lone adults and females with offspring during summer and winter, we used the timestamp of the photographic captures obtained from camera traps during 2018–2021. A non‐parametric kernel density function was fitted to the circular time data in order to produce the pairwise (females with offspring—lone adults) activity patterns and, subsequently, the coefficient of overlap (delta, Δ; values range between 0 (no overlap) and 1 (complete overlap)) was estimated from the area under those two different density functions (Linkie and Ridout 2011; Meredith and Ridout 2014; Ridout and Linkie 2009). We calculated Δ_4_ when the number of independent photographic detections of both states of sloth bears was > 75; otherwise, Δ_1_ was calculated in the case of < 75 detections (Linkie and Ridout 2011). We generated the 95% confidence interval using 10,000 bootstrapped samples of the overlap coefficient to check the precision of the estimates (Linkie and Ridout 2011). All analyses regarding the estimation of the overlap coefficient were carried out in the package ‘overlap’ (Meredith and Ridout 2021) implemented in R (R Core Team 2019).

The temporal overlap estimation for the activity of the lone adults and females with offspring may provide useful information regarding their broad, time‐bound interaction, but it is incapable of quantifying the proportion of time spent during different diel periods, in order to compare activities across the two groups. Hence, we further assessed whether or not such fine‐scale differences in activity patterns between the two groups of sloth bears exist, based on the newly developed Bayesian multinomial approach, using the package *Diel.Niche* (Gerber et al. 2024) in R (R Core Team 2019). Here, the diel period was divided into different phenotypes based on the animals' activity with the light availability, which are diurnal, nocturnal, crepuscular, and cathemeral (Anderson and Wiens 2017; Cox and Gaston 2024). However, cathemerality (active throughout the diel period) (Cox and Gaston 2024) remains much less explored in studies concerning activity patterns of organisms, but it is potent to understand the fine‐scale distributions of activity across the different diel phenotypes, which can be further classified as cathemeral general, crepuscular‐nocturnal, diurnal‐crepuscular, and diurnal‐nocturnal (Gerber et al. 2024). We considered lone adult bears and females with offspring, either nocturnal, crepuscular, or diurnal, based on a threshold activity probability of 0.80 for any of the respective phenotypes (Gerber et al. 2024). Similarly, the lower limit of the threshold activity probability was set at 0.10, equal to or above which the individual's activity will be differentiated into specific diel phenotype(s) (Gerber et al. 2024). Finally, we were interested in assessing the ‘selection’ of any specific diel phenotype by the two groups of sloth bears, so we compared the estimated proportion of time active (‘used’) and the total available time in proportion (‘available’) for any particular phenotype. The used/available > 1 indicates the selection, and < 1 indicates random choice or non‐selection. We refer to the documentation for the package *Diel.Niche* for further details (Gerber et al. 2024).

#### 2.3.3 Ethical Note

The fieldwork was carried out under the permit number Technical/4301/dated June 09, 2015, and issued by the Principal Chief Conservator of Forest (Wildlife Division), Madhya Pradesh, India. The data collection procedure was carried out entirely in a non‐invasive way by deploying automated motion‐sensored camera traps, following the standard protocol (Jhala et al. 2020). Due to their non‐invasive nature, camera trapping surveys were expected to photo‐capture sloth bears without disturbing or altering their movements.

## 3 Results

### 3.1 Camera Trap Detections

The camera traps were deployed in an average of 247 locations per season (min = 244, max = 254) and were active between 28 days (minimum) and 37 days (maximum), yielding a total effort of 47,893 trap nights (Table S2). During summer seasons, camera traps were active in 254 and 243 locations for 37 and 29 days, respectively (Table S2). Sloth bears (lone adults and females with offspring) were detected 2649 times during six seasons, yielding 441.5 detections per season (minimum 157, maximum 710) (Table S2). We found that 8.40% (*n* = 224) of the total number of bears detected in PTR were females with offspring (< 1 year and ≥ 1 year, i.e., yearlings), with 37.33 detections per season (Table S2). Females with offspring were detected more frequently (*n* = 131, ~58.5%) during two summer seasons than during four winters combined (*n* = 93, ~41.50%). About 29.7% (*n* = 76) and ~97% (*n* = 249) of the camera traps detected female sloth bears with offspring, and lone adults, respectively, at least once during the six seasons of the camera trap survey (2018–2021) (Figure S1).

### 3.2 Dynamic Multistate Occupancy

Sloth bears (lone adults) were detected more in camera trap locations where LST was less (*β* = −0.21 ± 0.04) (Table 1, Table S3, Figure S2); however, observations of females with offspring (both cubs and yearlings) were more prominent during summer (*β* = 0.46 ± 0.21) (Table 1, Table S3, Figure S2). The selected best detection models also contained uninformative parameters (95% confidence interval of *β* estimates overlapped zero) for *p*2 (Table 1 and Table S3). Detection probabilities (*p*1 and *p*2) and offspring observation probability (*δ*) varied between years, ranging from 0.09 to 0.43 (Table S4 and Figure S2). The observation probability of the offspring varied between 0.008 and 0.18, and the chance of only observing lone adult bears ranged from 0.08 to 0.24 (Table S4) when the true state was 2 for both scenarios. We also found that the difference between the probability of observing only offspring (*p*2 × *δ*) and lone adults (*p*2 × (1‐*δ*)) when the true state was 2 varied substantially during winter (16%–91%) compared to summer (20%–24%) (Table S4).

**TABLE 1:** Detection and observation probability parameter estimates (*β* estimates (standard error), and 95% confidence interval (lower confidence limit and upper confidence limit)) for the best detection models (ΔAIC < 2) for sloth bears, obtained from the dynamic multistate occupancy framework, in Panna Tiger Reserve, central India, during 2018–2021.

Parameters	Covariates	*β* estimates (SE)	95% CI LCL	95% CI UCL
*δ*	Summer	**0.46 (0.21)**	**0.05**	**0.87**
*p*1	LST_CT	**−0.21 (0.04)**	**−0.13**	**−0.29**
*p*2	Summer	0.15 (0.15)	−0.27	0.44
	Mean_days	−0.05 (0.15)	−0.24	0.34
	Winter	−0.04 (0.28)	−0.51	0.59

*Note:* Delta (δ)‐ Observation probability for females with offspring, *p*1 and *p*2—detection probabilities of bears when true state is 1 (occupied by lone adults) and 2 (occupied by females with offspring), respectively; Covariates considered: Summer‐ A categorical covariate denoting summer (typically from March–June) season, LST_CT‐ Mean (summer and winter) land surface temperature at camera trap location (30 m resolution), Winter‐ A categorical covariate denoting winter (typically from November–February) season, Mean_days‐ Mean number of days when camera traps were active at each location during 2018–2021. Bold values indicate statistically significant estimates, for which the 95% confidence intervals do not overlap zero.

**FIGURE 3:**
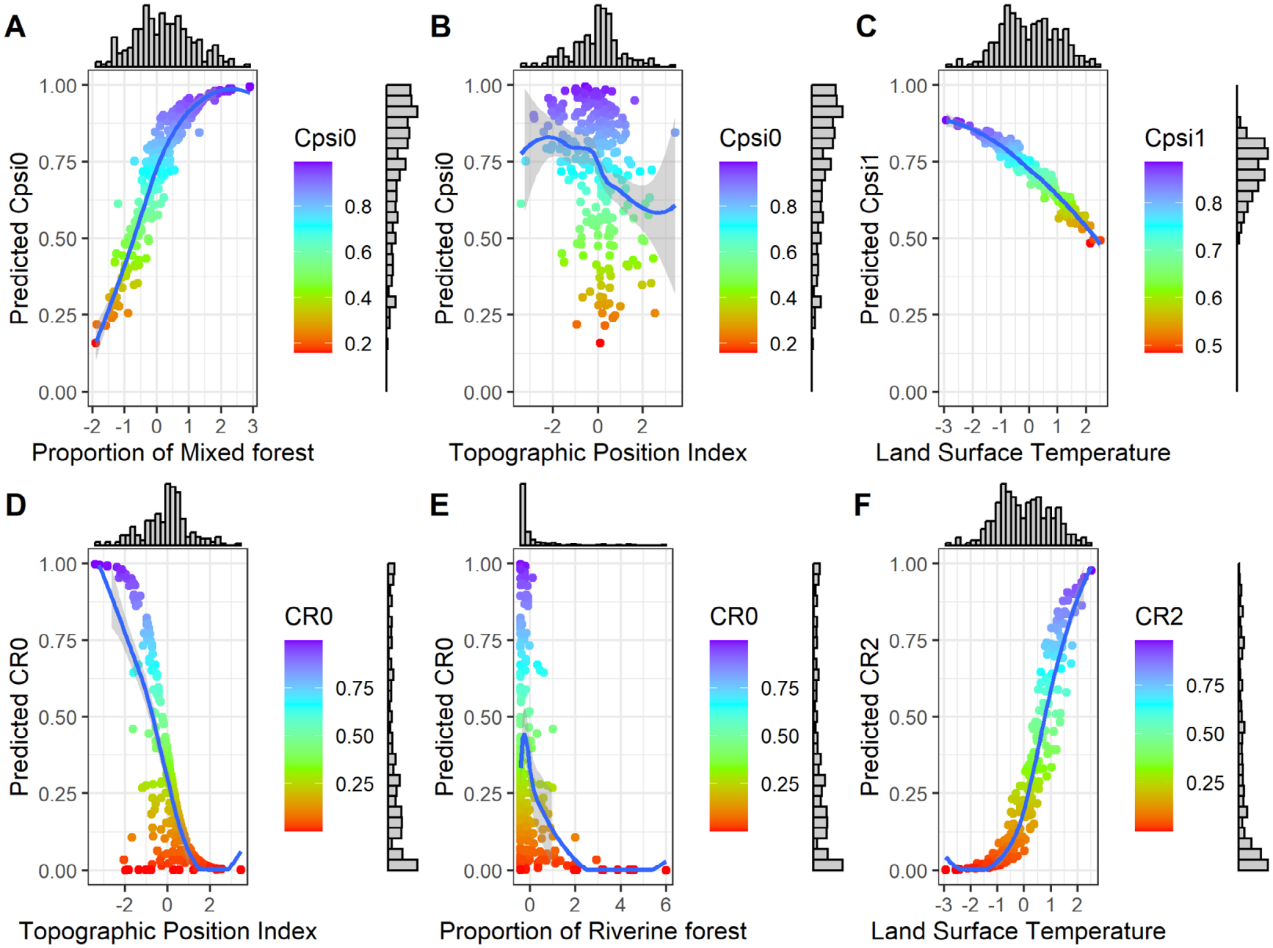
Effects of covariates on the occupancy of lone adult sloth bears (A–C) and females with offspring (D–F) in Panna Tiger Reserve, central India, during 2018–2021. Parameters estimated: Colonisation probability (CΨ0) of lone adult bears, consistent occupancy (CΨ1) of lone adult bears over the seasons (2019–2021), colonisation probability (CR0) from state 0 (none occupied) to 2 (occupied by females with offspring), and the consistent use (CR2) of a site by females with offspring over the seasons. Covariates considered: Proportion of mixed forest in 1 km circular buffer around camera trap location, Topographic position index (TPI), calculated around 1 km circular buffer from camera trap location, Land surface temperature (LST), mean value (summer and winter) calculated around 1 km circular buffer from camera trap location, Proportion of riverine forests in 1 km circular buffer around camera trap location. Covariate values are on a standardised (Z‐score) scale.

The top models (ΔAIC < 2), including the detection and observation covariates, indicated that forest types (mixed and riverine), terrain complexity (TPI), and temperature (LST) had effects on the dynamic occupancy of sloth bears (Table 2). During the initial season (winter 2018), occupancy of lone adults had a strong negative (*β* = −0.76 ± 0.23, 95% CI: 0.31–1.21) relationship with distance to villages. From the second season onwards (2019 winter to 2021 winter), the proportion of mixed forest, TPI and LST influenced transition occupancy probabilities (CΨ_0_ and CΨ_1_) for lone adults. Lone adult bears colonised valley areas (i.e., higher negative value of TPI; *β* = −0.33 ± 0.21, 95% CI: −0.08‐0.74) with higher proportion of mixed forest (*β* = 1.38 ± 0.34, 95% CI: 0.71–2.05), and consistently used areas with low LST (*β* = 0.39 ± 0.17, 95% CI: 0.06–0.72) (Tables 2 and 3; Figure 3). However, females with offspring colonised (CR0) in valley areas (*β* = −2.03 ± 0.73, 95% CI: −0.6–(−3.46)) and avoided riverine forests (*β* = −2.10 ± 1.32, 95% CI: −0.49‐4.67) when those areas were previously unoccupied by any bears. Furthermore, females with offspring consistently used (CR_2_) areas with higher LST (*β* = 1.98 ± 0.95, 95% CI: 0.12–3.84) (Tables 2 and 3; Figure 3). For the initial season, site use by at least one female with offspring was influenced by LST and the proportion of mixed forest, but those were uninformative.

**TABLE 2:** Dynamic multistate occupancy models for the occupancy probabilities of sloth bears during 2018–2021, in Panna Tiger Reserve, central India.

Model	AIC	ΔAIC	AICwqt	Model likelihood	No. Pars
Ψ(Vildist), CΨ(Mixed + TPI, LST,.), r(.), CR(TPI + Riverine,.,LST), δ(Summer), p(LST_CT,.)	8163.78	0.00	0.24	1.00	18
Ψ(Vildist), CΨ(Mixed,LST,.),r(LST), CR(TPI + Riverine,.,LST), *δ* (Summer),*p*(LST_CT,.)	8165.58	1.8	0.21	0.89	18
Ψ(Vildist), CΨ(Mixed,LST,.),r(Mixed), CR(TPI + Riverine,.,LST), δ(Summer),p(LST_CT,.)	8165.70	1.92	0.10	0.41	18
Ψ(Vildist), CΨ(Mixed,LST,.),r(TPI), CR(TPI + Riverine,.,LST), δ(Summer),p(LST_CT,.)	8166.07	2.29	0.08	0.36	18

*Note:* Parameters included: Occupancy (Ψ) of adult sloth bears in the initial year (Winter 2018), colonisation probability (CΨ_0_) and consistent occupancy (CΨ_1_) of lone adult bears over the seasons (2019–2021), colonisation probability (CR_0_) and the consistent use (CR_2_) of a site by female with offspring over the seasons, observation probability (δ) of offspring, detection probability of bears when true state is 1 (occupied by lone adults) (*p*1) and 2 (occupied by female with offspring) (p2). Occupancy covariates: Vildist: distance to the nearest village, Mixed‐ proportion of mixed forest in 1 km circular buffer around camera trap location, TPI‐ topographic position index, around 1 km circular buffer from camera trap location, LST‐ mean (summer and winter) land surface temperature around 1 km circular buffer from camera trap location, Riverine‐ proportion of riverine forests in 1 km circular buffer around camera trap location. Detection covariates: Summer‐ a categorical covariate denoting summer (March–June) season, LST_CT‐ mean (summer and winter) land surface temperature at camera trap location (30 m resolution), Winter‐ a categorical covariate denoting winter (November–February) season, Mean_days‐ mean number of days when camera traps were active at each location during 2018–2021, and Slope_30m‐ slope around the camera trap location (30 m resolution). Model selection was based on AIC (Akaike Information Criterion) values; best models were chosen based on ΔAIC < 2, and model weight (AICwqt), model likelihood and number of parameters (No. Pars) in the models were reported.

**TABLE 3:** Parameter estimates (*β* estimates (standard error), and 95% confidence interval (lower confidence limit and upper confidence limit)) for the best occupancy models (ΔAIC < 2) for sloth bears, obtained from the dynamic multistate occupancy framework, in Panna Tiger Reserve, central India, during 2018–2021.

Parameters	Covariates	*β* estimates (SE)	95% CI LCL	95% CI UCL
Ψ	Vildist	**−0.76 (0.23)**	**−0.31**	**−1.21**
CΨ_0_	Mixed	**1.38 (0.34)**	**0.71**	**2.05**
	TPI	−0.33 (0.21)	−0.08	0.74
CΨ_1_	LST	**−0.39 (0.17)**	**0.06**	**0.72**
*R*	LST	0.18 (0.24)	−0.29	0.65
	Mixed	−0.18 (0.28)	−0.37	0.73
	TPI	−0.05 (0.24)	−0.42	0.52
CR_0_	TPI	**−2.03 (0.73)**	**−0.60**	**−3.46**
	Riverine	−2.10 (1.32)	−0.49	4.67
CR_2_	LST	**1.98 (0.95)**	**0.12**	**3.84**

*Note:* Parameters included: Occupancy Ψ and R of lone adults and females with offspring, respectively, in the initial year (winter 2018); colonisation probability (CΨ_0_) and consistent occupancy (CΨ_1_) of lone adult bears over the seasons (2019–2021); colonisation probability (CR_0_) and the consistent use (CR_2_) of a site by females with offspring over the seasons. Covariates considered: Vildist‐ distance to the nearest village, Mixed‐ proportion of mixed forest in 1 km circular buffer around camera trap location, TPI‐ topographic position index, around 1 km circular buffer from camera trap location, LST‐ mean (summer and winter) land surface temperature, around 1 km circular buffer from camera trap location, Riverine‐ proportion of riverine forests in 1 km circular buffer around camera trap location. Bold values indicate statistically significant estimates, for which the 95% confidence intervals do not overlap zero.

### 3.3 Single‐Season Multistate Occupancy

During two summer seasons (2019 and 2020), the detection probabilities of sloth bears, i.e., *p*1 and *p*2 were significantly influenced by the effort of camera traps (mean number of days cameras were active) (*β* = 3.18 ± 1.09) and LST during summer seasons (*β* = −0.24 ± 0.06) in the camera trap locations (30 m resolution) (Tables 4 and 5; Figure S4). The LST also had a strong effect on observations of females with offspring (*β* = −0.27 ± 0.12) (Tables 4 and 5; Figure S3).

**TABLE 4:** Single‐season multistate occupancy models for the occupancy probabilities of sloth bears during the summer seasons of 2019 and 2020 in Panna Tiger Reserve, central India.

Model	AIC	ΔAIC	AICwqt	Model likelihood	No. Pars
Ψ (TPI + Scrub), *R* (Riverine + Bamboo), *δ* (LST_CT_Summer), *p*1 (Mean_days), *p*2 (LST_CT_Summer)	3356.72	0.00	0.33	1.00	12
Ψ (TPI + Mixed), *R* (Riverine + Scrub), δ (LST_CT_Summer), *p*1 (Mean_days), *p*2 (LST_CT_Summer)	3356.91	0.19	0.30	0.91	12
Ψ (TPI + Mixed), R (Riverine + TPI), *δ* (LST_CT_Summer), *p*1 (Mean_days), *p*2 (LST_CT_Summer)	3357.47	0.75	0.23	0.69	12
Ψ (TPI + Mixed), *R* (Riverine + Mixed), *δ* (LST_CT_Summer), *p*1 (Mean_days), *p*2 (LST_CT_Summer)	3358.40	1.68	0.14	0.43	12

*Note:* Parameters included: Occupancy (Ψ) of lone adult sloth bears (State 1), occupancy probability (*R*) of females accompanied by offspring (State 2), observation probability (*δ*) of offspring, detection probability of bears when the true state is 1 (*p*1) and 2 (*p*2). Occupancy covariates: TPI‐ Topographic position index around 1 km circular buffer from camera trap location, Scrub‐ proportion of scrub lands in 1 km circular buffer around camera trap location, Mixed‐ proportion of mixed forest in 1 km circular buffer around camera trap location, Riverine‐ proportion of riverine forests in 1 km circular buffer around camera trap location, and Bamboo‐ proportion of bamboo forests in 1 km circular buffer around camera trap location. Detection covariates considered: LST_CT_Summer‐ mean land surface temperature at camera trap location (30 m resolution) for summer seasons of 2019 and 2020, Mean_days‐ mean number of days when camera traps were active at each location during the summer seasons of 2019 and 2020. Model selection was based on AIC (Akaike Information Criterion) values; best models were chosen based on ΔAIC < 2, and model weight (AICwqt), odel likelihood, and number of parameters (No. Pars) in the models were reported.

**TABLE 5:** Parameter estimates (*β* estimates (standard error), and 95% confidence interval (lower confidence limit and upper confidence limit)) for the best detection models (ΔAIC < 2) for sloth bears, obtained from the single‐season multistate occupancy framework, in Panna Tiger Reserve, central India, during summers of 2019–2020.

Parameters	Covariates	*β* estimates (SE)	95% CI LCL	95% CI UCL
Ψ	TPI	**−0.65 (0.25)**	**0.16**	**1.14**
	Scrub	**−0.60 (0.23)**	**−0.15**	**−1.05**
	Mixed	**0.81 (0.31)**	**0.20**	**1.42**
*R*	Riverine	**−0.53 (0.23)**	**−0.08**	**−0.98**
	Bamboo	0.54 (0.35)	−0.15	1.23
	Scrub	0.44 (0.40)	−0.34	1.22
	TPI	−0.29 (0.29)	−0.28	0.86
	Mixed	−0.10 (0.32)	−0.53	0.73
δ	LST_CT_Summer	**−0.26 (0.12)**	**−0.02**	**−0.49**
*p*1	Mean_days	**3.18 (1.09)**	**1.04**	**5.32**
*p*2	LST_CT_Summer	**−0.25 (0.06)**	**−0.13**	**−0.37**

*Note:* Parameters included: Occupancy (Ψ) of lone adult sloth bears (State 1), occupancy probability (*R*) of females accompanied by offspring (State 2), observation probability (δ) of offspring, detection probability of bears when the true state is 1 (*p*1) and 2 (*p*2). Occupancy covariates: TPI‐ topographic position index, calculated around 1 km circular buffer from camera trap location, scrub– proportion of scrub lands in 1 km circular buffer around camera trap location, mixed– proportion of mixed forest in 1 km circular buffer around camera trap location, riverine– proportion of riverine forests in 1 km circular buffer around camera trap location, and bamboo– proportion of bamboo forests in 1 km circular buffer around camera trap location. Detection covariates considered: LST_CT_Summer‐ mean land surface temperature at camera trap location (30 m resolution) for summer seasons of 2019 and 2020, mean_days‐ mean number of days when camera traps were active at each location during summer seasons of 20,219 and 2020. Bold values indicate statistically significant estimates, for which the 95% confidence intervals do not overlap zero.

**FIGURE 4:**
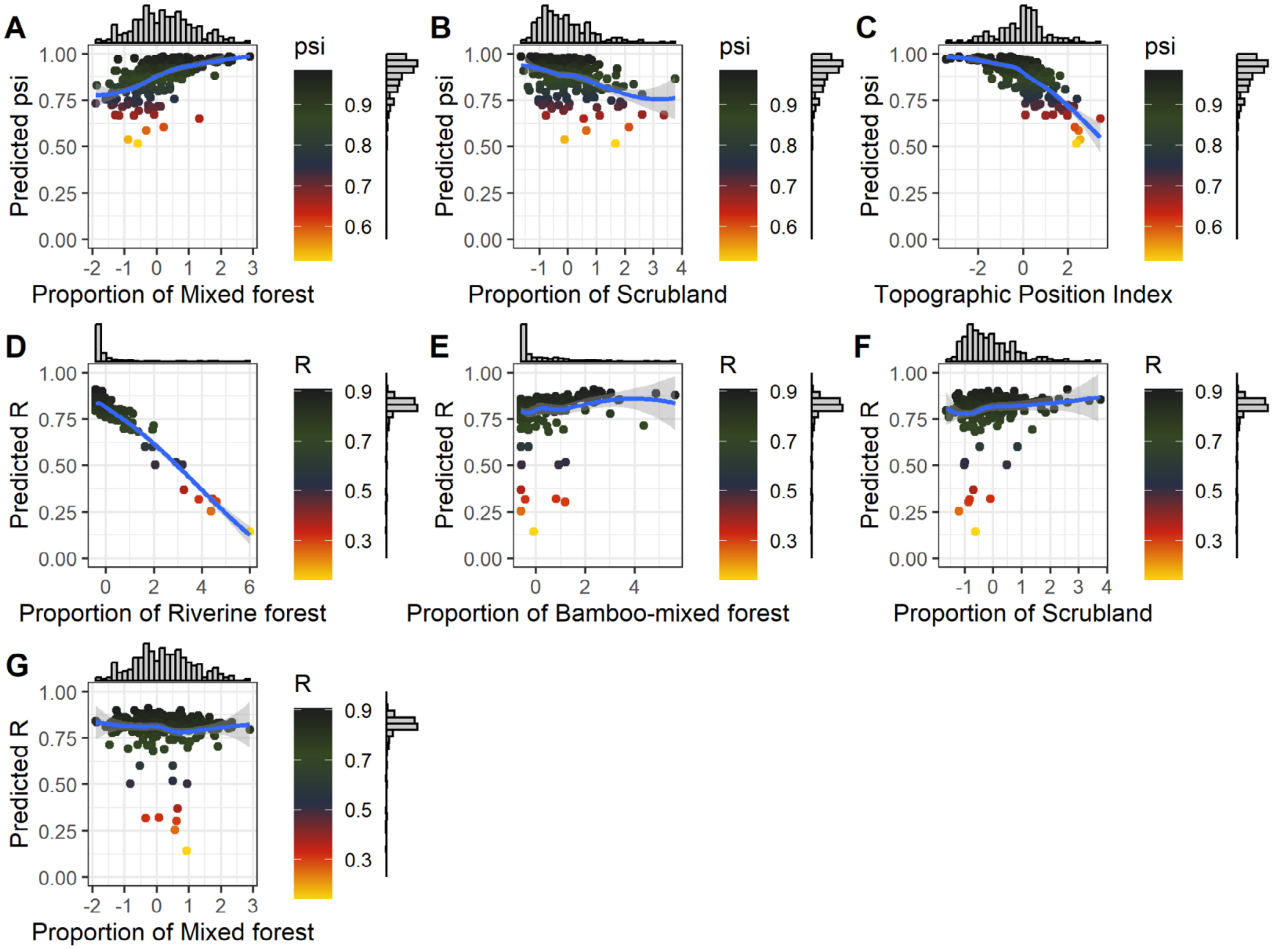
Effects of covariates on the occupancy of lone adult sloth bears (A–C) and females with offspring (D–G) in Panna Tiger Reserve, central India, during the summers of 2019 and 2020, obtained from single‐season multistate occupancy models. Parameters estimated: Occupancy probability (Ψ) of lone adult bears, and occupancy probability (*R*) of females with offspring. Covariates considered: Proportion of mixed forest in 1 km circular buffer around camera trap location, proportion of scrubland in 1 km circular buffer around camera trap location, Topographic position index (TPI), calculated around 1 km circular buffer from camera trap location, Proportion of Riverine forest in 1 km circular buffer around camera trap location, and proportion of bamboo‐mixed forest in 1 km circular buffer around camera trap location. Covariate values are on a standardised (*Z*‐score) scale.

With the best detection and observation covariates, as mentioned above, lone adult bears' occupancy was strongly influenced by the forest types (scrub lands (*β* = −0.60 ± 0.23), mixed forests (*β* = 0.81 ± 0.31)) and TPI (*β* = −0.65 ± 0.25), whereas the proportion of riverine forests (*β* = −0.53 ± 0.23) primarily predicted the occupancy of females with offspring (Tables 4 and 5; Figure 4). The best models also indicated the presence of other covariates (bamboo forests, scrub lands, mixed forests, and TPI) associated with the females' occupancy with offspring (Table 4), but those remained uninformative (Table 5; Figure 4).

### 3.4 Activity Patterns

**FIGURE 5:**
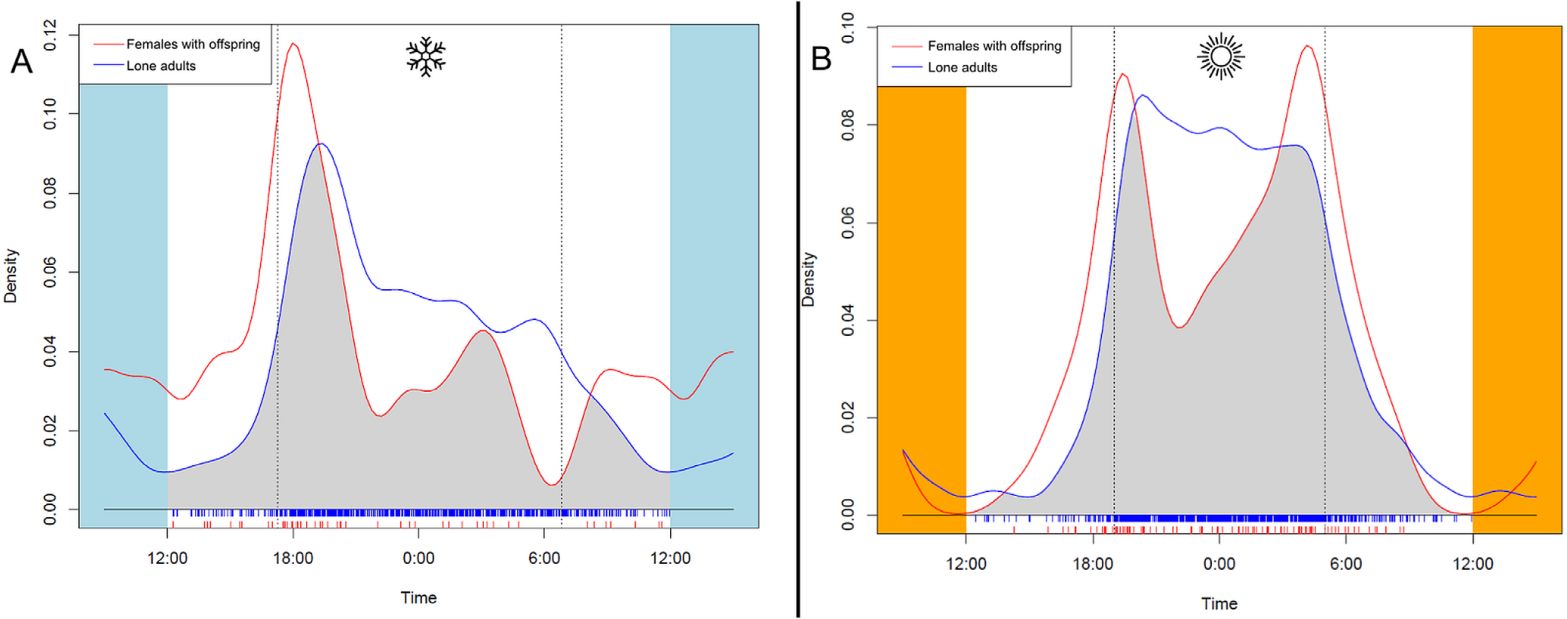
Activity overlaps (grey‐shaded area) between lone adult sloth bears and females with offspring (cubs and yearlings) during four winters from 2018 to 2021 (coefficient of overlap, i.e., Δ = 72%; A) and two summers from 2019 to 2020 (Δ = 72%; B), calculated from camera trap photographic captures, in Panna Tiger Reserve, central India.

Female sloth bears accompanied by offspring showed an apparent similar activity pattern with lone adults, characterised by high temporal overlaps in summer—81% (95% CI = 73%–89%), and winter −72% (95% CI = 61%–83%), respectively (Figure 5). However, the activity peak between the two differed slightly during summers (2019 and 2020) and winters (2018–2021) (Figure 5).

**FIGURE 6:**
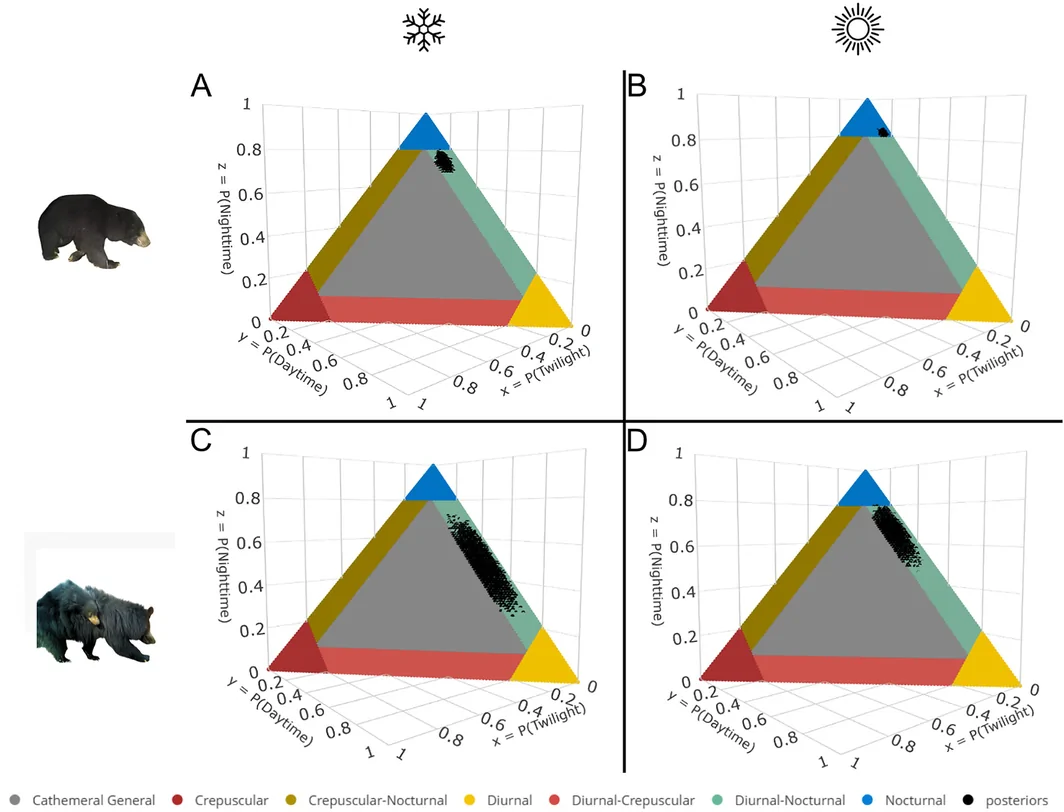
Posterior distribution of activity probability of lone adult sloth bears (A and B) and females with offspring (C and D) during winter (A and C) and summer seasons (B and D) from 2018 to 2021 in Panna Tiger Reserve, central India. Results were obtained from the ‘General’ hypothesis test, as described by Gerber et al. 2024; the points, situated in the coloured portions (different diel phenotypes, e.g., nocturnal and diurnal‐nocturnal, in this case) of the triangle, represented the corresponding hypothesis that was most supported by the samples.

Lone adults (median = 0.73, BCI = 0.70–0.76 in winter; median = 0.81, BCI = 0.80–0.83 in summer) and females with offspring (median = 0.48, BCI = 0.35–0.61 in winter; median = 0.66, BCI = 0.56–0.74 in summer) both showed an inclination towards nocturnal activities (Figure 6, Figure S4). However, females with offspring had a significant proportion (> 0.10) of total activity during the daytime for both seasons, but especially in the winter when they were as active (median = 0.46, BCI = 0.33–0.59) (Figure S4) in the daytime as in the night, indicating a diurnal‐nocturnal diel phenotype (Figure 6). We found that lone adults showed a stronger selection of nocturnality (used_nocturnal_/Available_nocturnal_ > 1) in both seasons, compared to females with offspring (Table S5). Similarly, females with offspring also showed selection for nocturnal activity in both seasons, but used daytime as a proportion to its availability (used_diurnal_/Available_diurnal_ = 1) during winter (Table S5).

## 4 Discussion

We documented the first‐ever evidence of spatial segregation among sloth bears with respect to their demographic states related to breeding biology. Throughout the seasons, females with offspring (cubs and yearlings) showed a marked spatial avoidance by preferring areas that lone adults use less. The multi‐season multistate models enabled us to understand the seasonal dynamics of the occupancy probability for lone adults and breeding females as a function of environmental covariates, while accounting for the detection and observation probabilities for adults and offspring. Furthermore, during the summer seasons, females with cubs (< 1 year old) did not seem to have a strong relationship with most of the environmental covariates, but still showed some segregation with lone adults. However, their temporal activity patterns also showed fine‐scale segregation, irrespective of seasons.

### 4.1 Occupancy of Lone Adult Individuals

In this study, the lone adults or single individuals usually colonised areas where a combination of specific forest types, terrain complexity and land surface temperature is persistent across the seasons. In the Indian Subcontinent, sloth bears are forest‐dwelling species but can occur in different land use types (Puri et al. 2015). However, they generally show an affinity towards certain forest types at local scales, which is possibly related to forage opportunity (Joshi et al. 1997). In central Indian landscapes (including PTR), sloth bears frequently used mixed or miscellaneous forest (comprised of miscellaneous tree species) habitats, possibly due to the availability of fruiting trees and colonial insects (Akhtar et al. 2004; Ramesh et al. 2012; Chaudhuri, Rajaraman, et al. 2022; Yoganand 2005), a pattern also observed for lone adults in the present study. Being a tropical bear species, sloth bears do not hibernate but take shelter in natural cavities, crevices, and dense shrubs situated in rocky escarpments or rugged terrain habitats during the daytime to avoid ambient thermal stress, as well as for parturition during winter (Akhtar et al. 2007; Chaudhary et al. 2025; Swaminathan et al. 2023; Yoganand 2005). Sloth bears prefer rugged landscapes wherever available and accessible, which is evident from many parts of their distribution, including central India (Jangid et al. 2023; Puri et al. 2015, 2023; Rot et al. 2023). Consistently, adult sloth bears in PTR also colonised areas with lower TPI values, i.e., the valley areas surrounded by plateaus with ridges that supposedly provide day resting and maternal den sites (Yoganand 2005). The relationship between sloth bear occurrence and terrain complexity was not significant (*p* > 0.05), but it indicated they may use other terrain types as well, such as flat plateau tops with higher TPI values. Interestingly, lone adults consistently used sites with lower land surface temperatures across the seasons in PTR. The physiological effect of heat stress is evident in many mammals, including carnivores (Hansen 2009; Rabaiotti and Woodroffe 2019) and bears as well (Rogers et al. 2020). To cope with heat stress, ursids would preferably show behavioural adaptations, such as reducing and/or shifting activity peaks, preference for higher‐elevation areas, and using habitat features that can provide shade (Rogers et al. 2020; Schneider et al. 2020). Given their high exposure to ambient temperature, tropical bears are likely to use such shade‐providing microhabitats frequently to mitigate the heat stress (Schneider et al. 2020). Similar to such findings, our results also indicated behavioural adaptations shown by adult bears, by preferring areas with low annual temperature, as well as being strictly crepuscular and nocturnal, which (the latter) was known previously in the central Indian landscapes and other parts of the Indian Subcontinent (Akhtar et al. 2007; Chaudhuri, Bandyopadhyay, et al. 2022; Joshi et al. 1999; Ramesh et al. 2013; Yoganand 2005). Since our study was conducted in a broadly undisturbed area (core zone) of PTR, the effect of human pressure in the study area was least expected. Still, we found adult bears occurred in areas close to the villages during the first season (winter 2018) of the sampling, but by and large, not in the subsequent seasons (at least from the best models). As evident from other studies, sloth bears are known to exploit food resources from human‐dominated landscapes, depending on the seasonal variation of food availability (Bargali et al. 2004; Desai et al. 2026; Palei et al. 2020; Prajapati et al. 2021). Furthermore, phenological changes or fruiting failures in a particular season may cause bears to venture into human‐dominated areas in search of food. Such resource availability‐driven changes in annual and seasonal diet occur in temperate ursids (Naves et al. 2006; Stenset et al. 2016), but are poorly understood in sloth bears and remain a subject for future research.

### 4.2 Occupancy of Females With Offspring and Spatio‐Temporal Segregation

The occupancy of female sloth bears accompanied by offspring during the initial season remained uninformative, that is, the predictors included in the top‐ranked models were too weak to explain the variation in their occupancy. However, across the seasons, female bears with offspring (cubs and yearlings) showed a strong preference for the terrain complexities, corresponding to the lower TPI values, especially in those sites that were not occupied by lone adult bears previously. Corroborating our findings, past studies also indicated that female bears generally prefer to give birth to young ones in secure dens or cavities or crevices (maternal dens), most of which are located on steep slopes and rocky escarpments (Akhtar et al. 2007; Chaudhary et al. 2025; Swaminathan et al. 2023; Yoganand 2005). However, after spending ~3 months (pre‐parturition to post‐parturition) in the maternal dens, females emerge with cubs but continue to use dens on a daily basis for day‐resting (Joshi et al. 1999; Swaminathan et al. 2023; Yoganand 2005). Hence, such habitats will be of key importance for females with offspring, irrespective of age classes (cubs and yearlings), as reflected in our results. While empirical evidence remains limited, other than maternal dens, resting dens serve as a means of a thermoregulatory strategy adopted by sloth bears (Akhtar et al. 2007). Likewise, the present study also found lone adults using similar habitats for day resting purposes, indicating a species‐level rather than individual‐level (females with offspring vs. lone adults, in this case) choice to manage the thermal stress. However, the strongly significant relationship (compared to a relatively weak predictor for lone adults) between females with offspring and TPI perhaps explained the more intensive use of such habitats in PTR. In mammals, reproductive females, both pregnant and lactating, produce an increased endogenous heat (Speakman and McQueenie 1996). The reproductive female sloth bears, hence, are possibly in greater need of exerting thermoregulatory behaviour compared to lone adults, which was also evident in brown bears' spatial distribution (Rogers et al. 2020). On the contrary, results from summer seasons' breeding occupancy showed a weak relationship (*p* > 0.05) between females with cubs and TPI, whereas a significant (*p* < 0.05) one for lone adults, indicating possible segregation during high heat stress. During summer, lone adults perhaps occupied most of the available denning habitats, and females with cubs were forced to choose habitats with less terrain complexity to avoid possible agonistic confrontation with adult bears. Intriguingly, females with cubs also consistently used areas with high LST across the seasons, something in contradiction with lone adult bears and thermoregulatory behaviour shown by other ursids (Rogers et al. 2020; Schneider et al. 2020). As discussed previously, occupying warmer habitats may impose a greater physiological cost for all the bears, irrespective of reproductive class. Such behaviour could be a straightforward strategy to avoid the cooler areas occupied by lone adults. In PTR, the repeated use of warmer habitats possibly drives females with offspring to utilise the valleys and rugged terrains more frequently for day resting, to dissipate the extra heat produced during lactation or raising cubs (Swaminathan et al. 2023; Yoganand 2005). Female sloth bears accompanied by their cubs avoided the riverine forests in PTR, especially during the summer seasons. Riverine forests are characterised by trees and shrubs alongside perennial and seasonal streams, but the latter form the majority of this forest type. However, riverine forests around the perennial water sources also attract other mammals (including large carnivores) across the seasons, but especially during the pinch periods. Females with cubs probably avoided such crowded areas in order to ensure the safety of the offspring, as indicated by our findings from the summer seasons. Previous studies have reported such behaviours in other bear species while sharing resources with sympatric carnivores (Ordiz et al. 2020). For sloth bears, Joshi et al. (1999) also suspected possible spatial and temporal segregation between female sloth bears with cubs and large carnivores. However, lone adults exhibited distinct patterns in preferring certain habitat/forest types during summer. They continued to use valleys and areas with a higher proportion of mixed forests, likely for day resting and foraging on fruiting trees, respectively. No other covariates strongly explained the occupancy of females with offspring, although the direction (either positive or negative) of the occupancy‐covariate relationship or choosing different habitats altogether indicated some avoidance. Additionally, to the best of our understanding, we cannot rule out the possibility of an apparent non‐avoidance or random movement of females accompanied by cubs with respect to single adults. Reproductive bears may not always avoid potential infanticidal conspecifics and could use areas irrespective of the presence of unrelated adults. Such behaviour likely reflects limited habitat availability or a lower site‐specific risk of infanticide, which is highly dependent on the population dynamics (Norton et al. 2018; Penteriani et al. 2020). As opposed to our expectations, we could not find strong evidence that either females with cubs or lone adults used cooler areas during summer. In addition, the broad activity patterns of lone adults and females with offspring did not differ in either of the seasons. Their proportion of time active during a day (24 h) also showed little difference in summer (females with cubs were slightly more active during daytime, compared to lone adults), indicating, by and large, no apparent limitation in their activity during summer due to high thermal stress. Summer is the fruiting season in most parts of the Indian Subcontinent, and fruits dominate the diet of sloth bears (Bargali et al. 2004; Joshi et al. 1997; Ramesh et al. 2009; Rather et al. 2020; Yoganand 2005) during this season. When the food sources are highly seasonal, bears' activity (irrespective of different reproductive classes) may not always be restricted due to high ambient temperatures, and they could remain more active in food acquisition, which was observed in both temperate and tropical bears (Hwang and Garshelis 2007; McLellan and McLellan 2015).

The activity patterns of lone adults and females with offspring were mainly nocturnal regardless of season, which aligns with previous findings (Chaudhuri, Bandyopadhyay, et al. 2022; Ramesh et al. 2013; Yoganand 2005). However, contrary to our expectation, the Bayesian multinomial approach revealed that females with offspring extended their activities during the daytime (diurnal‐nocturnal activity pattern) in comparison to the lone adults in both seasons, but especially in winter. Such a temporal shift could be possible either due to avoiding other unrelated bears or large predators (e.g., tigers and leopards) that may threaten offspring survival (Joshi et al. 1999). We are not certain whether females with offspring modified their activity due to the presence of large predators; still, our study indicated adult conspecifics could influence the temporal activity patterns of such breeding individuals, in addition to their spatial segregation. Extended daytime activity may facilitate females with offspring to access the resource‐rich areas that are mainly occupied by lone individuals when the latter remain least active. The novelty of this Bayesian multinomial framework allowed us to precisely differentiate the seasonal usage of diel periods by different demographic states, while reducing the ambiguity arising from conventional qualitative and visual interpretation of activity patterns. These fine‐scale avoidance behaviours are not very well‐documented in solitary, elusive species like sloth bears; hence, future research should focus on such complex behavioural aspects for further understanding.

The multistate occupancy models separately estimate detection and observation probabilities as a function of relevant covariates, which is essential for the correct estimates and interpretation of occupancy parameters (Bailey et al. 2007; Mackenzie et al. 2009; Nichols et al. 2007). It is worth noting that the ability to tease apart the occupancy and detection process in hierarchical models, even as a function of the same covariates, could be of great benefit and can unravel interesting ecological patterns (Kéry 2008). For less territorial species like sloth bears, multistate models explicitly treat a site as occupied by state 2 individuals even when it contains detections of state 1 individuals, which may theoretically lead to underestimation of state 1 individuals. However, given our objectives of assessing changes in occupancy (colonisation and persistence), the dynamic framework allows the site states to vary across seasons and reduces the likelihood of such bias arising from static state assignment. Additionally, as expected, we found substantially higher detections (~70% of the total number of sites) and occupancy of lone adults than females with offspring, across all seasons, and the detection of lone adults was not influenced by the detections of females with offspring. Single adult sloth bears were more frequently detected in camera trap locations with lower LST, consistent with their occupancy in cooler areas across the seasons. However, the observation probability of offspring was positively biassed during summer, as summer coincides with the emergence period of females with cubs (< 1 year) from their natal dens after parturition, which yielded more detections of offspring in camera traps. Interestingly, during summer, the observation probability of offspring and the detection probability of any adults or females with offspring when the site was in state‐2 were dependent on the low LST around the camera trap locations. Such detection events probably indicated an underlying fine‐scale mechanism shown by the breeding individuals for avoiding high temperatures during summer despite their occupation of high LST areas, to circumvent the confrontation with potentially infanticidal conspecifics. The LST did not influence the detection of lone adults, but the detection probability sharply increased with the camera trap effort, i.e., the number of days camera traps were active during summer seasons. Such a relationship was common in camera trap studies but could be species‐ and site‐specific (O'Connor et al. 2017). The multistate occupancy models, corrected for detection and observation probability of different states, enabled us to precisely infer the differential site use by lone adult bears and females accompanied by offspring for different habitats. While a few studies incorporated the demographic states in other ursids (Evans et al. 2024; Fisher et al. 2014; Sultaire et al. 2023), our study further extended this approach to the aspects of behavioural ecology in sloth bears, thus representing a novel application in this context.

### 4.3 Probable Cost to Females With Offspring

The habitat use by female sloth bears with their offspring in open, hotter, and more complex terrain is perhaps a fitness trade‐off to sub‐optimal habitats to ensure cub survival from potential infanticide and to avoid resource competition. Although the infanticide‐mediated response is not clear, the pattern of avoidance shown by females with offspring over the seasons could come with a physiological cost, which has been demonstrated in other organisms, including mammalian carnivores (Ben‐David et al. 2004; Brown and Kotler 2004; Lima and Dill 1990; Verdolin 2006). The repeated use of higher‐temperature areas across seasons and the reduced availability of high‐quality den sites, especially during summers, could negatively affect the fitness of lactating females due to thermal constraints. In support of our findings, this phenomenon has also been reported in brown bears (Rogers et al. 2020). Extending activity during daylight hours, probably to avoid confrontation with lone adults (and large predators) and reduce resource competition, may also pose additional thermal stress to the females with offspring. Furthermore, the occupancy of habitats (e.g., mixed forests) with fruiting trees and insect colonies by lone adults may have precluded the females with offspring from occupying those habitats and forced them to inhabit poor‐quality, open habitats (scrublands), leading to a possible constraint on their foraging and overall fitness outcome. Unless diet and nutritional quality are monitored, we cannot be certain about the nutritional status of such individuals. Still, based on the habitat types and characteristics used by females with offspring, it is possible that these habitats might not be the best quality ones in terms of foraging opportunities. Additionally, long‐term research on ursids showed that differential resource selection by breeding females to minimise either intraspecific competition or NPI can exert a population‐level effect through changes in fecundity (Mattson and Reinhart 1995; Wielgus et al. 2001; Wielgus and Bunnell 2000) or imposing an additional cost to the female reproductive system (Stearns 1989). Nonetheless, the probable impact of such spatio‐temporal segregation on the physiological fitness of female bears with offspring is a matter of concern and is a subject for future research.

## 5 Conclusion

In the present study, demographic states emerged as an important driver of variation in space use and activity patterns among sloth bears in the PTR. This interesting aspect, with significant linkage to fitness outcomes provides a basis for incorporating demography‐specific behaviours into understanding the energy expenditure of female bears with offspring to offset the environmental limiting factors. Whether it is a physiological mechanism that allows for energy saving or alternative resources in a poor‐quality habitat would provide a more nuanced understanding of sloth bears' ecology. Our findings have powerful implications for future habitat and conflict management activities; for instance, differential habitat use and activity patterns of lone individuals and females with offspring may result in varied levels of exposure to human‐dominated landscapes, which can influence the conflict patterns. While taking account of gaps in our study, that is, incorporating effects of predators and human exposure, we suggest future studies should focus on the nutritional and stress ecology of breeding females based on individual‐level genetic identification and fine‐scale resource selection measures. Overall, these findings underscore the importance of recognising demographic states in fine‐scale behavioural patterns shown by sloth bears, and highlight the utility of approaches that can capture such complexity.

## Author Contributions

**Sankarshan Chaudhuri:** conceptualization (lead), formal analysis (equal), investigation (equal), methodology (lead), software (equal), validation (equal), visualization (equal), writing – original draft (lead), writing – review and editing (equal). **Supratim Dutta:** data curation (lead), formal analysis (equal), investigation (equal), methodology (equal), software (equal), visualization (equal), writing – original draft (supporting), writing – review and editing (equal). **Ramesh Krishnamurthy:** conceptualization (equal), funding acquisition (lead), investigation (equal), methodology (equal), project administration (lead), resources (lead), supervision (lead), validation (equal), writing – review and editing (equal).

## Funding

This work was supported by the National Water Development Agency, WII/KR/PROJECT/PLMP/2017‐18/F(1). National Tiger Conservation Authority, WII/KR/PROJECT/PTRP/2013‐14/012(B).

## Conflicts of Interest

The authors declare no conflicts of interest.

## Supporting information


**Data S1:** Supporting Information.

**Figure S1:** Photographic captures of lone adult sloth bears or non‐breeding individuals (A) and females with offspring or breeding individuals (B), obtained from camera trap surveys from 2018 to 2021 in Panna Tiger Reserve, central India.
**Figure S2:** Detection probability (p1) of lone adult sloth bears as a function of land surface temperature (LST) at camera trap locations (30 m resolution) (A), and probability of observing the sloth bear offspring (δ, i.e., delta; offspring includes cubs and yearlings) in the camera trap photographs while the site is occupied by females with cubs (state 2) (B), obtained from the dynamic multistate occupancy models, in Panna Tiger Reserve, central India, during 2018–2021. Seasons include Winter (January–February) of 2018, Winter (January–February) of 2019, summer (May–June) 2019, Winter (December 2019–February 2020) 2020, summer (May–June) 2020 and winter (December 2020–February 2021) 2021. The scale of the covariate (Land surface temperature at camera trap location) shown here was *z*‐transformed.
**Figure S3:** Effects of covariates on *p*1 detection probability of lone adult sloth bears; (A), *p*2 (detection probability of females with offspring and or lone adults, when the site is occupied by females with offspring; (B), and delta (δ), observation probability of offspring while the site is occupied by female with offspring; (C), in Panna Tiger Reserve, central India, during the summers of 2019 and 2020, obtained from single‐season multistate occupancy models. Covariates considered: Mean number of days when camera traps were active at each location during the summer seasons of 2019 and 2020, and Land Surface Temperature (mean value) at camera trap location (30 m resolution) for the summer seasons of 2019 and 2020. Covariates values are on a standardised (*Z*‐score) scale.
**Figure S4:** Probability of activity of lone adult sloth bears and females with offspring in three major diel phenotypes (Twilight, Daytime and Nighttime activities) during winter and summer seasons in Panna Tiger Reserve, central India. The data obtained from camera trap detections from 2018 to 2021. The points represented the proportion of activity in three diel phenotypes, with 95% Bayesian confidence intervals.
**Table S1:** List of covariates for occupancy, observation, and detection probabilities of sloth bears (both lone adults and females with offspring) in Panna Tiger Reserve, central India, during 2018–2021.
**Table S2:** Details of camera traps deployed from 2018 to 2021, including the effort (trap‐nights **±** standard error (SE)) and photographic captures of sloth bears (lone adults and females with offspring, and all individuals) in Panna Tiger Reserve, central India.
**Table S3:** Dynamic multistate occupancy models for the detection probability (p) of sloth bears when the true state is 1 (sites used by adult lone bears) and 2 (sites used by females with offspring) and the probability of observing females with offspring (δ) upon detection at sites of state 2 (while holding occupancy parameters constant), during 2018–2021, in Panna Tiger Reserve, central India. Covariates considered: Summer‐ a categorical covariate denoting summer (March–June) season, LST_CT‐ Land surface temperature at camera trap location (30 m resolution), Winter‐ a categorical covariate denoting winter (November–February) season, Mean_days‐ Mean number of days when camera traps were active at each location during 2018–2021, and Slope_30m‐Slope around the camera trap location (30 m resolution). Model selection was based on AIC (Akaike Information Criterion) values; best models were chosen based on ΔAIC < 2, and model weight (AICwqt), Model likelihood and number of parameters (No. Pars) in the models were reported.
**Table S4:** Year‐wise detection probability estimates (*p*1 and *p*2 with standard errors (SE)) when sites are occupied by adult lone bears (*p*1) and by females with offspring (*p*2), and observation probability (δ with standard errors (SE)) for offspring given the site is occupied by females with offspring, obtained from dynamic multistate occupancy models, in Panna Tiger Reserve, central India, during 2018–2021. While the true state of a site is 2, that is, the site is occupied by females with offspring, *p*2 × δ indicated the observation probability of offspring, and *p*2 × (1‐δ) indicated the probability of observing only adult lone bears. Seasons included 2018_W‐ Winter (January–February) of 2018, 2019_W‐ Winter (January–February) of 2019, 2019_S‐ summer (May–June) of 2019, 2020_W‐ Winter (December 2019–February 2020) of 2020, 2020_S‐ summer (May–June) of 2020 and 2021_W‐ winter (December 2020–February 2021) of 2020.
**Table S5:** Diel phenotype selection for lone adult sloth bears (non‐breeding) and females with offspring (breeding) based on proportion of time spent active (used) with respect to time availability during twilight, day and night in Panna Tiger Reserve, central India.

## Data Availability

The data associated with this study are available as Supporting Information.
