# Supplementary material for: Loners Have It: Insights on Breeding and Non‐Breeding Individuals of Sloth Bears in Central India
Source: Ecol Evol. 2026 Sep 17;16(9):e74231. doi: 10.1002/ece3.74231 (PMC13585475; doi:10.1002/ece3.74231)
Supplement: Supplementary file 1 [file ECE3-16-e74231-s001.docx]

**Supplementary Table 1.** List of covariates for occupancy, observation, and detection probabilities of sloth bears (both lone adults and females with offspring) in Panna Tiger Reserve, central India, during 2018-2021.

| **Covariates** | **Description** | **Range of values** |
| --- | --- | --- |
| **Occupancy covariates** |  |  |
| Topographic Position Index (TPI) | TPI determined the terrain complexity of an area (e.g., valley, flat, ridges, moderate slopes) and was calculated using the elevation of each cell and subtracting the mean elevation of the neighboring cell (Weiss 2001), and the mean values were extracted for 1km buffer around camera trap locations by using the Spatial Analyst tool in ArcGIS 10.2. | -11.18 – 10.74 |
| Slope (degree) | The slope was calculated by the Spatial analyst Surface tool, and mean values were extracted for each 1km buffer around camera trap locations in Arc GIS 10.2. | 2.63 – 14.44 |
| Tree cover canopy (in %) | Obtained from Global 2010 Tree Cover, Global Land Analysis & Discovery (GLAD) (Hansen et al. 2013). Mean values of tree cover canopy were extracted for each 1km buffer around camera trap locations in Arc GIS 10.2. | 0 – 29.75 |
| Distance to village (km) | Euclidean distance to the nearest village from the camera trap location was calculated using the Spatial analyst tool in Arc GIS 10.2. | 0.55 – 6.109 |
| Distance to water source (km) | Euclidean distance to the nearest water source (natural or artificial) from the camera trap location was calculated using the Spatial analyst tool in Arc GIS 10.2. Due to the seasonal changes, we calculated water availability separately for the winter and summer seasons. | 0.08 – 5.27 (mean value for winter and summer)  Summer: 0 – 5.51 |
| Proportion of forest types | Forest type map of PTR was processed from Sentinel-2 data (10m resolution), comprised of 11 classes, i.e., water bodies, scrubland, grassland, non-forest, trees outside forest, *Anogeissus pendulla* forest, bamboo mixed forest, mixed forest, riverine forest, teak forest and teak-mixed forest, using ERDAS IMAGINE 9.2 (Leica Geosystems, St. Gallen, Switzerland). The proportion of area (in km^2^) of each forest type was calculated and extracted for each 1km buffer around camera trap locations in Arc GIS 10.2. | Scrubland: 0.02 – 0.29  Grassland: 0.004 – 0.58  *A. Pendulla* forest: 0 – 0.32  Bamboo mixed forest: 0 – 0.24  Mixed forest: 0.12 – 0.85  Riverine forest: 0 – 0.22  Teak forest: 0 – 0.45  Teak mixed forest: 0 – 0.71 |
| Land surface temperature (LST)  (in ^o^C) | Land Surface Temperature is the radiative skin temperature of the land surface, as measured in the direction of the remote sensor (Becker and Li 1990). Remotely sensed land surface temperature was prepared by using the thermal infrared, near-infrared, and red bands, specifically bands 10, 5, and 4, respectively. For the summer and winter seasons of 2019, Landsat 8 images were separately processed (30m resolution) using ERDAS IMAGINE 9.2 (Leica Geosystems, St. Gallen, Switzerland). A step-by-step method was followed by calculating the Top of Atmospheric (ToA) spectral radiance, ToA to brightness temperature conversion, determining land surface emissivity, and subsequently emissivity corrected LST was calculated for winter and summer (Avdan and Jovanovska 2016). The mean LST values were extracted for each 1km buffer around camera trap locations in Arc GIS 10.2. | 27.76 – 33.88 (mean value for winter and summer)  Summer season: 33.17 – 40.71 |
| **Detection covariates** |  |  |
| Camera trap effort (No. of days) | The mean number of days camera traps were operational, was calculated from each site, for six seasons (2018 winter, 2019 winter, 2019 summer, 2020 winter, 2020 summer, and 2021 winter) and two seasons (2019 summer and 2020 summer) | 11 – 36 (mean no. of days for all the seasons)  Summer: 14 – 36 |
| Season – summer and winter | The climatic season when the camera trapping survey was carried out, the summer season lies between March and June; the winter season lies between November and February. | - |
| Land surface temperature (LST)  (in ^o^C) | The mean LST value was obtained from 30m around the camera trap location in Arc GIS 10.2, i.e., the exact pixel value where the camera trap was deployed. | 26.07 – 34.41 (mean value for winter and summer)  Summer: 28.23 – 41.76 |
| Slope (degree) | The slope value was obtained from 30m around the camera trap location in Arc GIS 10.2, i.e., the exact pixel value where the camera trap was deployed. | 0 – 25 |
| Tree cover canopy (in %) | Mean values of the tree cover canopy were extracted for each 30m buffer around camera trap locations in Arc GIS 10.2, i.e., the exact pixel value where the camera trap was deployed | 0 – 45 |
| Detections of females with offspring | Total number of photographic captures/camera trap site of females with offspring obtained during six seasons (2018-2021) of camera trapping surveys | 0-4 |

**Supplementary Table 2.** Details of camera traps deployed from 2018 to 2021, including the effort (trap-nights **±** standard error (SE)) and photographic captures of sloth bears (lone adults and females with offspring, and all individuals) in Panna Tiger Reserve, central India.

|  | **2018** | **2019** | | **2020** | | **2021** |
| --- | --- | --- | --- | --- | --- | --- |
|  | **Winter (January-February)** | **Winter (January-February)** | **Summer (May-June)** | **Winter (December-March)** | **Summer (May-June)** | **Winter (December-February)** |
| **No. of camera trap locations** | 244 | 248 | 254 | 245 | 243 | 249 |
| **Mean Trap-nights± SE** | 27.57**±0.39** | 31.70**±0.37** | 37.13**±0.24** | 32.30**±0.47** | 29.05**±0.47** | 29.33**±0.53** |
| **Total trap-nights** | 7058 | 8116 | 9505 | 8269 | 7437 | 7508 |
| Total no. of sloth bears | 293 | 157 | 710 | 442 | 689 | 358 |
| No. of adult lone sloth bears | 278 | 149 | 625 | 401 | 643 | 329 |
| No. of female sloth bears with offspring | 15 | 8 | 85 | 41 | 46 | 29 |

**Supplementary Fig.1.** Photographic captures of lone adult sloth bears or non-breeding individuals (A) and females with offspring or breeding individuals (B), obtained from camera trap surveys from 2018 to 2021 in Panna Tiger Reserve, central India.

**Supplementary Table 3.** Dynamic multistate occupancy models for the detection probability (p) of sloth bears when the true state is 1(sites used by adult lone bears) and 2 (sites used by females with offspring) and the probability of observing females with offspring (δ) upon detection at sites of state 2 (while holding occupancy parameters constant), during 2018-2021, in Panna Tiger Reserve, central India. Covariates considered: Summer- a categorical covariate denoting summer (March-June) season, LST_CT- Land surface temperature at camera trap location (30m resolution), Winter- a categorical covariate denoting winter (November-February) season, Mean_days- Mean number of days when camera traps were active at each location during 2018-2021, and Slope_30m-Slope around the camera trap location (30m resolution). Model selection was based on AIC (Akaike Information Criterion) values; best models were chosen based on ΔAIC <2, and model weight (AICwqt), Model likelihood and number of parameters (no.Pars) in the models were reported.

| **Model** | **AIC** | **ΔAIC** | **AICwqt** | **Model Likelihood** | **no.Pars** |
| --- | --- | --- | --- | --- | --- |
| δ(Summer), p(LST_CT,.) | 8221.98 | 0.00 | 0.42 | 1.00 | 11 |
| δ(Summer), p(LST_CT, Summer) | 8223.03 | 1.05 | 0.25 | 0.59 | 12 |
| δ(Summer), p(LST_CT, Mean_days) | 8223.85 | 1.87 | 0.17 | 0.39 | 12 |
| δ(Summer), p(LST_CT, Winter) | 8223.95 | 1.97 | 0.16 | 0.37 | 12 |
| δ(Summer), p(Slope_30m, Summer) | 8242.44 | 20.46 | 0.00 | 0.00 | 12 |

**Supplementary Fig. 2.** Detection probability (p1) of lone adult sloth bears as a function of land surface temperature (LST) at camera trap locations (30m resolution) (**A**), and probability of observing the sloth bear offspring (δ, i.e., delta; offspring includes cubs and yearlings) in the camera trap photographs while the site is occupied by females with cubs (state 2) (**B**), obtained from the dynamic multistate occupancy models, in Panna Tiger Reserve, central India, during 2018-2021. Seasons include Winter (January-February) of 2018, Winter (January-February) of 2019, summer (May-June) 2019, Winter (December 2019-February 2020) 2020, summer (May-June) 2020, and winter (December 2020-February 2021) 2021. The scale of the covariate (Land surface temperature at camera trap location) shown here was z-transformed.

**Supplementary Table 4.** Year-wise detection probability estimates (p1 and p2 with standard errors (SE)) when sites are occupied by adult lone bears (p1) and by females with offspring (p2), and observation probability (δ with standard errors (SE)) for offspring given the site is occupied by females with offspring, obtained from dynamic multistate occupancy models, in Panna Tiger Reserve, central India, during 2018-2021. While the true state of a site is 2, i.e., the site is occupied by females with offspring, p2 x δ indicated the observation probability of offspring, and p2 x (1-δ) indicated the probability of observing only adult lone bears. Seasons included 2018_W- Winter (January-February) of 2018, 2019_W- Winter (January-February) of 2019, 2019_S- summer (May-June) of 2019, 2020_W- Winter (December 2019-February 2020) of 2020, 2020_S- summer (May-June) of 2020, and 2021_W- winter (December 2020-February 2021) of 2020.

| **Seasons** | **p1 (SE)** | **p2 (SE)** | **δ (SE)** | **p2 x δ** | **p2 x (1-δ)** |
| --- | --- | --- | --- | --- | --- |
| 2018_W | 0.43 (0.02) | 0.43 (0.02) | 0.43 (0.02) | 0.18 | 0.24 |
| 2019_W | 0.09 (0.01) | 0.09 (0.01) | 0.09 (0.009) | 0.008 | 0.08 |
| 2019_S | 0.29 (0.02) | 0.29 (0.02) | 0.39 (0.05) | 0.11 | 0.18 |
| 2020_W | 0.20 (0.02) | 0.20 (0.01) | 0.22 (0.02) | 0.04 | 0.15 |
| 2020_S | 0.30 (0.02) | 0.30 (0.02) | 0.40 (0.05) | 0.12 | 0.18 |
| 2021_W | 0.19 (0.02) | 0.20 (0.01) | 0.19 (0.01) | 0.04 | 0.16 |

**Supplementary Fig. 3.** Effects of covariates on p1 (detection probability of lone adult sloth bears; A), p2 (detection probability of females with offspring and or lone adults, when the site is occupied by females with offspring; B), and delta (δ, observation probability of offspring while the site is occupied by female with offspring; C), in Panna Tiger Reserve, central India, during the summers of 2019 and 2020, obtained from single-season multistate occupancy models. Covariates considered: Mean number of days when camera traps were active at each location during the summer seasons of 20219 and 2020, and Land Surface Temperature (mean value) at camera trap location (30m resolution) for the summer seasons of 2019 and 2020. Covariates values are on a standardized (Z-score) scale.

**Supplementary Fig. 4.** Probability of activity of lone adult sloth bears and females with offspring in three major diel phenotypes (Twilight, Daytime and Nighttime activities) during winter and summer seasons in Panna Tiger Reserve, central India. The data obtained from camera trap detections from 2018 to 2021. The points represented the proportion of activity in three diel phenotypes, with 95% Bayesian confidence intervals.

**Supplementary Table 5.** Diel phenotype selection for lone adult sloth bears (non-breeding) and females with offspring (breeding) based on proportion of time spent active (used) with respect to time availability during twilight, day, and night in Panna Tiger Reserve, central India.

|  | **Lone adults** | | | | **Females with offspring** | | | |
| --- | --- | --- | --- | --- | --- | --- | --- | --- |
|  | **Winter** | | **Summer** | | **Winter** | | **Summer** | |
|  | **Used time**  **(in proportion)** | **Available time (in proportion)** | **Used time (in proportion)** | **Available time (in proportion)** | **Used time (in proportion)** | **Available time (in proportion)** | **Used time (in proportion)** | **Available time (in proportion)** |
| **Twilight** | 0.06 | 0.11 | 0.02 | 0.12 | 0.06 | 0.11 | 0.06 | 0.12 |
| **Day** | 0.21 | 0.46 | 0.18 | 0.56 | 0.46 | 0.46 | 0.28 | 0.56 |
| **Night** | 0.73 | 0.43 | 0.79 | 0.31 | 0.48 | 0.43 | 0.66 | 0.31 |
